# The complete mitochondrial genome of *Phoenicurus frontalis* (Passeriformes: Muscicapidae)

**DOI:** 10.1080/23802359.2020.1773332

**Published:** 2020-06-05

**Authors:** Feng-jun Li, Lu Qiao, Yi-jie Yang, Kong Yang, Nan Yang, Bi-song Yue

**Affiliations:** a College of Life Sciences, Sichuan University, Chengdu, P. R. China; b Batang County Forestry and Grassland Bureau, Batang, P. R. China; c Institute of Qinghai-Tibetan Plateau, Southwest Minzu University, Chengdu, P. R. China

**Keywords:** *Phoenicurus frontalis*, complete mitochondrial genome, evolutionary relationships

## Abstract

The Blue-fronted Redstart *Phoenicurus frontalis* (Muscicapidae) belongs to the family Muscicapidae, distributed in central China, Qinghai-Tibet plateau and the Himalayas. The conservation status of this species is Least Concern (LC) in IUCN. In this study, the complete mitogenome of *P. frontalis* was determined. The mitogenome is a circular molecule of 16,776 bp in length, containing 13 protein-coding genes, 2 ribosome RNA genes, 22 transfer RNA genes, and 1 non-coding region. We reconstructed a phylogenetic tree based on Bayesian inference for 15 Passeriformes species. The new mitogenome data would provide useful information for application in conservation genetics and further clarify the phylogenetic evolution of this species.

The Blue-fronted Redstart *Phoenicurus frontalis* (Muscicapidae) belongs to the family Muscicapidae, the Old World flycatchers (Ali and Ripley 1983). Its range includes the northern regions of the Indian Subcontinent and parts of Southeast Asia. It is found in Bhutan, India, Laos, Myanmar, Nepal, Thailand, Tibet and Vietnam. The species is associated with the temperate forests. The female is brownish-grey, with paler underparts (Guo and Zhang 2015). The conservation status of this species is Least Concern (LC) in IUCN. In China, the species also has been listed as a Least Concern (LC) species by the red list of China’s vertebrates (Jiang et al. 2016). Up to now, no any complete mitochondrial genome data of *P. frontalis* is available in the GenBank. In this study, we sequenced the complete mitochondrial genome of *P. frontalis* (GenBank number: MT360379) and examined its phylogenetic position with other Passeriformes species.

The tissue samples were obtained from Chaqing Songduo Nature Reserve, Baiyu County, Sichuan Province, P. R. China (Latitude: 30.984°N, Longitude: 99.342°E, Altitude: 3,808 m), and maintained in Sichuan University, Chengdu. The stored number of the sample is CQSD-014. Total genomic DNA was extracted from liver tissue using the DNA extraction kit (Aidlab Biotech, Beijing, China). The mitochondrial genomes of *P. auroreus* (NC_026066.1) is used to design primers for polymerase chain reaction (PCR) and used as template for gene annotation.

The total complete mitogenome sequence of *P. frontalis* is 16,776 bp, which is composed of 13 protein-coding genes (PCGs), 2 ribosome RNA genes, 22 transfer RNA genes and, 1 non-coding region (D-Loop). The total base composition of the *P. frontalis* mt genome is an A + T-rich pattern of the vertebrate mitochondrial genomes. ATG is the most common start codon, GTG is used for COX1. *Phoenicurus frontalis* had one non-coding region: a 1212 bp control region (D-loop). The major noncoding control region (D-loop) had none tandem repeat elements.

The phylogenetic relationship for the newly determined mitochondrial genome sequences was examined with those of 15 Muscicapidae and 1 outgroup species. The BI analysis was performed using BEAST v1.7 (Drummond et al. 2012), and the best-fit model (GTR + I + G) of nucleotide evolution was selected using the AIC test in JModelTest 2 (Darriba et al. 2012). The phylogenetic tree resulting from the Bayesian inference (BI) analyses showed that *P. frontalis* was a sister to *P. auroreus* (posterior probability =1.00) (Figure 1).

**Figure 1.**
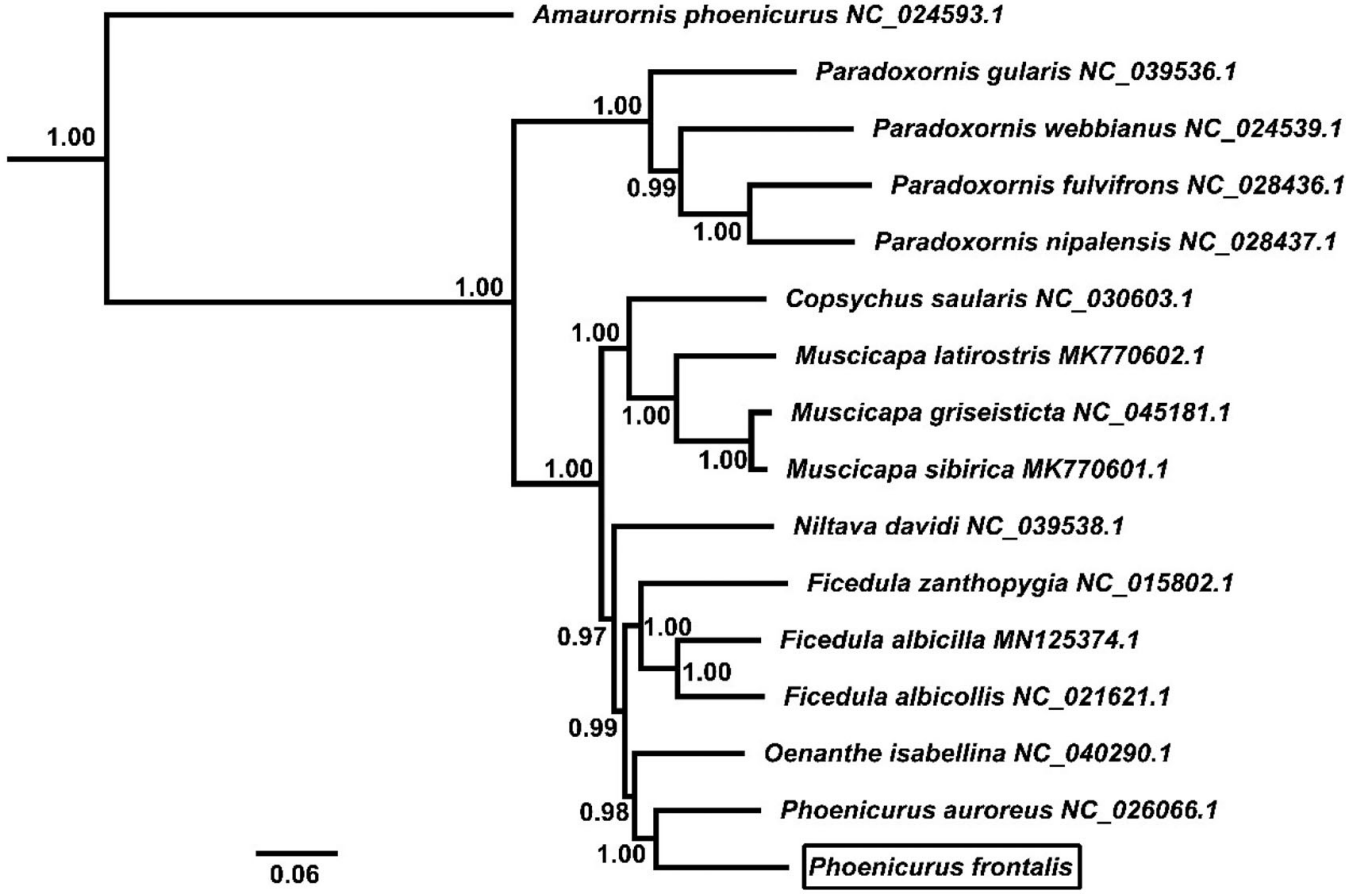
Phylogenetic tree derived from 12 protein-coding gene sequences from 16 complete mitochondrial genomes using BI analysis. Numbers by the nodes indicate Bayesian posterior probabilities.

This study is the first one to report and analyze the complete mitochondrial genome of *P. frontalis*. The data will contribute to solve the phylogenetic relations of the genus *Phoenicurus*, would provide reference information for further study of this species and serve as molecular tools to protect it.

## Data Availability

The data that support the findings of this study are available from the corresponding author, Nan Yang, upon reasonable request. The data are openly available in GenBank of NCBI at https://www.ncbi.nlm.nih.gov, reference number MT360379.
